# Profound Muscle Weakness and Pain after One Dose of Actonel

**DOI:** 10.1155/2009/693014

**Published:** 2009-10-11

**Authors:** Irina Badayan, Merit E. Cudkowicz

**Affiliations:** Neurology Clinical Trials Unit, Massachusetts General Hospital, Harvard Medical School, Charlestown, MA 02129, USA

## Abstract

The World Health Organization (WHO) defines osteopenia as a bone density between 1 and 2.5 standard deviation (SD) below the bone density of a normal young adult Iqbal 2000. Osteoporosis is defined as 2.5 SD or more below that reference point Iqbal 2000. Bisphosphonates are a group of medications used to treat osteoporosis, Padget's disease of bone, and osteopenia. We report a woman who developed profound muscle weakness and pain after one dose of Risedronate (Actonel).

## 1. Introduction

Risedronate (Actonel) is a bisphosphonate used for the treatment of osteopenia that inhibits bone resorption via actions on osteoclasts or osteoclast precursors, leading to an increase in bone mineral density. Pharmacologic therapy for osteopenia is recommended as early as possible to prevent the greater bone loss [1]. The most common side effects of Risedronate include fever and flu-like symptoms, hypocalcemia, bone and joint pain, peripheral edema, fatigue, change in bowel movements, osteonecrosis of the jaw, and atrial fibrillation [2]. We describe a case report of severe weakness as a potential new side effect of Risedronate in a woman with osteopenia.

## 2. Case Report

A 53-year-old Caucasian woman presented with diffuse muscle weakness and pain. Past medical history was notable for gastroesophageal reflux disease, diverticulitis, intermittent lower back pain, and osteopenia. She took one dose of Risedronate, 35 mg for osteopenia. Within 15 hours, she developed severe aching muscle pain (10/10 on Visual Analog Scale) throughout the body with fevers, sweats, chills, tingling throughout the body, and loss of bladder control. In the Emergency Room, she was told she had a drug reaction from Risedronate and prescribed oxycontin for pain management. Evaluation included normal complete blood counts and electrolytes. Potassium and creatinine were mildly low at 3.4 mEq/L, and 0.5 mg/dL, respectively. The CO2 was elevated at 31.4 mEq/L (normal range 22.0–29.0 mEq/L), Calcium, creatine phosphokinase (CPK) and ESR were normal. Neurological examination in the emergency room was notable for decreased strength in proximal lower extremities graded at 4−/5 using the Medical Research Council (MRC) scale. Deep tendon reflexes were normal. The only medications patient was taking at the time of the event were Nexium 40 mg per day and Posture D vitamins.

Over the next three weeks, she developed severe muscle cramps, bilateral lower extremity edema, and progressive muscle weakness and could not raise her arms above her head. She had several falls and became wheelchair bound. Diffuse pain (8/10 on VAS) in the bones and muscles of her limbs and back was present.

She underwent electromyography (EMG) testing which demonstrated mild bilateral median neuropathies bilaterally and active denervation (fibrillations and positive sharp waves) only in the right deltoid muscle. MRI of cervical spine was normal. Methylpresnisolone, 24 mg with a six day taper was prescribed without clinical benefit.

One month post dose, she had proximal weakness in the upper and lower extremities with bilateral deltoid graded at MRC 3−/5, hip flexors at 3−/5, and hamstrings at 4/5. Proximal muscle weakness was evident on gait testing. She was admitted to an outside hospital. During her stay, the work up for following differential diagnosis was done: multiple myeloma, Gullian-Barré syndrome, chronic inflammatory demyelinating polyneuropathy. Systemic Lupus Erythematosus, Waldestrom's macroglobulinemia, Cushing disease, myasthenia gravis, hyperparathyroidism, and polymyalgia rheumatica. Work up included an MRI of brain with and without gadolinium, a lumbar puncture for cerebrospinal fluid analysis, and a whole body positron emission tomography scan, all which were normal. MRI of the muscle was not performed. Results of a second EMG study were similar to the previous test with the exception that the active denervation changes in right deltoid were absent. A muscle biopsy was considered at the beginning of the hospitalization, but was deferred because her aldolase and repeat CPK tests were normal. She started a program of physical and occupational therapy, and acupuncture.

The pain and weakness began to slowly subside approximately four months after the dose. By six months postdose, she improved to the point that she no longer needed a cane to ambulate. One year postdose, she reports easy fatigability, severe pain (7/10 on VAS) in the left biceps and back, and mild weakness in her legs. She has difficulty going up the stairs and standing from a lying position. Neurological examination was notable for mild proximal lower extremity and hand weakness with MRC grades of 4+ iliopsoas, 5− abductor pollicis brevis, 4+ abductor digiti minimi, and 4+ first dorsal interosseous bilaterally with some weakness on the finger extensions. No atrophy or fasciculations were seen.

Two and a half years after taking one pill of Risedronate, the patient continues to improve. She reports some weakness of her shoulders, difficulty carrying heavy things, and some shortness of breath with exertion. Recently, she underwent quantitative muscle testing (QMT) to asses her muscle weakness [3]. Isometric strength of ten muscle groups was measured bilaterally. Her percent of predicted strength values indicated extensive weakness throughout, with muscle strength of different muscle groups ranging from 31.5% to 76.4%, with the mean strength 50.5%.

A MedWatch form was filed with the FDA.

## 3. Discussion

We describe a 53-year-old woman who developed a severe pain and weakness after taking one pill of Risedronate for treatment of osteopenia. The side effect of medication was so profound that patient lost ability to ambulate and two and a half years later is still not completely recovered.

According to 2004 Surgeon General report on bone health and osteoporosis, 33.6 million individuals over age 50 have low bone mass or osteopenia of the hip and are at risk of osteoporosis and its potential complications. The prevalence of osteoporosis and low bone mass is expected to increase to 12 million cases of osteoporosis and 40 million cases of low bone mass among individuals over the age of 50 by 2010, and to nearly 14 million cases of osteoporosis and over 47 million cases of low bone mass in individuals over that age by 2020 [4]. Bisphosphonates are approved for treatment of Paget's disease of bone, osteoporosis, and osteopenia.

Absorption of Risedronate after the oral dose is relatively rapid (Tmax 1 hour). There is no evidence of systemic metabolism of Risedronate with 80% of the drug excreted with urine and 20% absorbed by bone. Bioavailability of Risedronate sodium is poor (0.54%–0.75%) and half-life is 480 hours. Approximately half of the absorbed dose is excreted in urine within 24 hours [2].

Risedronate was used in the treatment of the patient presented in this case report. The FDA has received six serious adverse event reports of severe bone, joint, or muscle pain for this medication [5]. Pain (described as “incapacitating,” “disabling”) and loss of ability to climb stairs, ambulate, and perform the usual activities were reported as side effects of a different bisphosphonate, Alendronate (Fosamax), which may suggest a possible class effect [5]. According to the FDA article published in 2006, many patients underwent numerous diagnostic tests with mostly normal findings [5, 6]. Details on the exact number of patients and the names of diagnostic tests patients performed are not provided. There have not been other reported cases of proximal muscle weakness after Risedronate use.

Symptoms experienced by the patient described in this case report (rise in temperature and accompanying flu-like symptoms) resemble an acute phase response. The mechanism for this response in general appears to be associated with the release of tumor necrosis factor (TNF)*α*, interferon *γ* (INF-*γ*), interleukin 1 (IL1), and interleukin 6 (IL-6) although the effector cells that release these cytokines and the mechanism of action remains not completely understood [7]. Aminobisphosphonates (including Risedronate) increase serum levels of proinflammatory cytokines [8]. Therefore, it is possible that patient's muscle weakness might be an inflammatory response to the drug toxicity. Such hypothesis cannot be supported by patient's levels of proinflammatory cytokines because they were not measured during the course of the disease. Muscle biopsy was not performed in this case, however will be an essential test to perform in similar cases to better assess etiology of symptoms. Pain and muscle weakness as adverse events of Risedronate may be underreported and not taken into consideration because of the subjective nature of pain, confusion with the pain from osteoporosis, and attribution of the loss of ambulation to pain. Combined with normal diagnostic tests, it may result in failure to recognize this potential side effect and a delay in stopping the bisphosphonate. Four of the bisphospohonates are listed in the Top 200 Brand Drugs By Units in 2007 accounting for almost 31 million prescriptions [9]. Given the number of patients receiving bisphosphonate therapy and the paucity of reports, the incidence of the complex of symptoms is assumed to be relatively low. However, the formal reporting of adverse drug events is known to understate their true incidence [10, 11].

For each patient, decisions regarding the therapy must also take into consideration the long period of treatment, substantial costs, and potential side effects. At this point, the risk factors and incidence of the incapacitating muscle pain and profound muscle weakness are unknown. Additional studies are needed to investigate the possible mechanism of pain, muscle weakness, and edema following bisphosphonate treatment. Even if low, the awareness of these side effects is very important because of the serious nature of the problem.
